# GC–MS analysis and pharmacological evaluations of *Phoenix sylvestris* (Roxb.) seeds provide new insights into the management of oxidative stress and hyperglycemia

**DOI:** 10.1002/fsn3.3196

**Published:** 2022-12-21

**Authors:** Md. Shafiullah Shajib, Shanta Islam, Safaet Alam, Ridwan Bin Rashid, Mirola Afroze, Mala Khan, Bidyut Kanti Datta, Lutfun Nahar, Satyajit Dey Sarker, Mohammad A. Rashid

**Affiliations:** ^1^ School of Biomedical Science, Faculty of Health Queensland University of Technology Queensland Brisbane Australia; ^2^ Department of Pharmacy Stamford University Bangladesh Dhaka Bangladesh; ^3^ Drugs and Toxins Research Division, BCSIR Laboratories Rajshahi, Bangladesh Council of Scientific and Industrial Research Rajshahi Bangladesh; ^4^ Department of Pharmaceutical Chemistry, Faculty of Pharmacy University of Dhaka Dhaka Bangladesh; ^5^ Department of Pharmacy State University of Bangladesh Dhaka Bangladesh; ^6^ Bangladesh Reference Institute for Chemical Measurements (BRICM), Bangladesh Council of Scientific and Industrial Research (BCSIR) Dhaka Bangladesh; ^7^ Laboratory of Growth Regulators, Palacký University and Institute of Experimental Botany The Czech Academy of Sciences Olomouc Czech Republic; ^8^ School of Pharmacy and Biomolecular Sciences Liverpool John Moores University Liverpool UK

**Keywords:** antihyperglycemic, antioxidant, Arecaceae, GC–MS, *Phoenix sylvestris*, traditional medicine

## Abstract

*Phoenix sylvestris* Roxb. (Arecaceae) seeds are used in the treatment of diabetes in the traditional system of medicine. The present study evaluated antihyperglycemic and antioxidant activities as well as the total phenolic and flavonoid content of the methanol extract of *P. sylvestris* seeds (MEPS). The constituents of the extract were identified by GC–MS analysis. MEPS demonstrated strong antioxidant activity against 2,2‐diphenyl‐1‐picrylhydrazyl (DPPH) (IC_50_ = 162.70 ± 14.99 μg) and nitric oxide (NO) (IC_50_ = 101.56 ± 9.46 μg/ml) free radicals. It also possesses a substantial amount of phenolics and flavonoids. It significantly (*p* < .05) reduced blood glucose levels in glucose‐loaded and alloxan‐induced diabetic mice at the doses of 150 and 300 mg/kg b.w., respectively. A total of 46 compounds were detected and identified by gas chromatography–mass spectroscopy (GC–MS) analysis, among which 8‐methylisoquinoline N‐oxide (32.82%) was predominant. The phytochemical study by GC–MS revealed that the MEPS possesses compounds which could be related to its antidiabetic and antioxidant activities. To recapitulate, *P. sylvestris* seeds can be a very good option for antidiabetic and antioxidant activity though further studies are still recommended to figure out the responsible phytochemicals and establish their exact mechanism of action.

## Graphical abstract

*Phoenix sylvestris* seeds are used in the treatment of diabetes in traditional system of medicine. The present study evaluated antihyperglycemic and antioxidant activities as well as total phenolic and flavonoid content of methanol extract of *P. sylvestris* seeds. (MEPS). The constituents of the extract were also identified by GC–MS analysis.

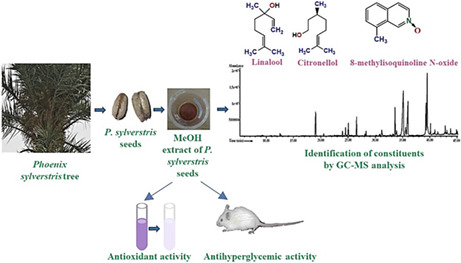

## 1 INTRODUCTION

Diabetes mellitus is the metabolic syndrome of the human body manifested by chronic hyperglycemia along with impaired metabolism of carbohydrates, protein, and fats due to diminished insulin secretion and/or action (Alam et al., 2022; Nayak & Roberts, 2006). Chronic hyperglycemia exacerbates the antioxidant action by increasing oxidative stress and reactive oxygen species (ROS) in islets of the pancreas (Savu et al., 2012). Furthermore, it has been reported that diabetes is responsible for the excess generation of free radicals due to the reduction of antioxidant levels in the body (Ali & Agha, 2009). Multiple antihyperglycemic agents along with insulin are currently available in the market, but they are not devoid of significant undesirable side effects (Pari & Saravanan, 2004). Recently, the use of plants and plant materials has attracted the attention of researchers for the development of new antihyperglycemic due to their promising efficacy and limited toxicity (Rates, 2001). In addition, antioxidants derived from plants have been shown to play important roles in improving diabetes‐associated disorders (Rahimi et al., 2005).

*Phoenix slylvestris* (L.) Roxb., a plant of the palm family Arecaceae, is commonly known as “Khejur” in Bangladesh. The plant seeds have been reported to be bacteriostatic against Gram‐positive and Gram‐negative organisms (Kothari, 2011). They are used in the treatment of dysentery, ague, and diabetes in the traditional medicine system (Beg & Singh, 2015; Ghani, 1998). Although traditional use advocates the use of *P. sylvestris* as a candidate for treating diabetes, no scientific report exists to corroborate this claim. Therefore, the present study aimed to determine the antioxidant action, total phenolic and flavonoid contents, and antihyperglycemic activity of seeds of *P. sylvestris* for the first time. The constituents of seed extract have also been identified by gas chromatography‐mass spectroscopic (GC–MS) analysis so that future researchers can find a nifty clue to identify responsible phytochemicals from the plant seeds to discover and develop novel therapeutics against diabetes and oxidative stress.

## 2 MATERIAL AND METHODS

### 2.1 Plant materials and extraction

The fully matured fruits of *P. sylvestris* were collected from Akabpur, Mainamati, Comilla, Bangladesh in July 2013. The fruits were identified by the authorities of Bangladesh National Herbarium, Mirpur, Dhaka, Bangladesh, and a voucher specimen has been deposited (accession no: DACB: 38499) for future reference. The seeds of *P. sylvestris* were separated from the fruits, dried, and ground to a coarse powder using a mechanical grinder. About 500 g of powdered seeds was mixed with 1200 ml of methanol (MeOH). The mixture was occasionally stirred and kept at 25 ± 2°C for 72 h. The extract was then filtered through the Whatman filter paper, number 41. The solvent was removed by using a rotary evaporator under reduced pressure at 40°C temperature and 50 rpm. Finally, 12.4 g (2.48% yield) concentrated extract was obtained, which was used for phytochemical and biological studies.

### 2.2 Chemicals and drugs

Chemicals and reagents used in this study were ‐ MeOH, 1,1‐diphenyl‐2‐picrylhydrazyl (DPPH), Griess reagent, quercetin, gallic acid, ascorbic acid, pentobarbital sodium (Sigma Co.), sodium carbonate (Na_2_CO_3_), Na‐K tartrate, aluminum chloride (AlCl_3_), Folin–Ciocalteu's reagent (Merck Co.), alloxan monohydrate (Loba Chemie Pvt. Ltd.). Metformin hydrochloride was obtained as a gift sample from Square Pharmaceuticals Ltd.

### 2.3 Ethical statements

The protocols for the current study were endorsed by the Ethics Committee of Stamford University Bangladesh (SUB/IAEC/13.05). The animals were treated according to the guidelines provided by The Swiss Academy of Medical Sciences and Swiss Academy of Sciences. After the experiments, animals were euthanized using pentobarbital sodium following the AVMA Guiding Principles for the Euthanasia of animals: 2013 edition. Necessary steps were taken to minimize animal suffering.

### 2.4 Phytochemical analysis

#### 2.4.1 Preliminary screening

MEPS was qualitatively screened for the detection of carbohydrates, reducing sugars, steroids, alkaloids, proteins, saponins, tannins, and flavonoids following the standard procedures (Ghani, 1998).

### 2.5 GC–MS (gas chromatography–mass spectroscopy) analysis

GC–MS analysis of the MeOH extract of *P. sylvestris* seeds was performed using Agilent 7890A (Agilent Technologies) capillary gas chromatograph interfaced to a 5975C inert XL EI/CI triple‐axis mass detector. The gas chromatograph was equipped with an HP‐5MSI fused capillary column of 5% phenyl, 95% dimethyl‐poly‐siloxane (film: 0.25 μm, length: 90 m, and diameter: 0.250 mm). The parameters of GC were programmed as follows: inlet temperature: 250^°^C; oven temperature; 90^°^C at 0 min raised to 200^°^C for 2 min (3^°^C/min) then 280^°^C for 2 min (15^°^C/min); carrier gas (Helium) flow rate: 1.1 ml/min; auxiliary temperature: 280^°^C. Total retention time for the chromatographic analysis was 46 min. The MS parameters were set as follows: quad temperature: 150°C; source temperature: 230°C; mode: scan mode; mass range: 50–550 *m/z*. The “NIST‐MS Library” was used for mass spectra analysis and identification of compounds. The relative percentage of separated compounds was determined from the peak areas of the total ionic chromatogram.

### 2.6 Determination of total phenolic content (TPC)

The total phenolics present in the MEPS were quantified using Folin–Ciocalteu's reagent (Singleton et al., 1999). An aliquot (0.5 ml) of Folin–Ciocalteu's reagent was taken and mixed with 1 ml of (200 μg/ mL) MEPS. After 5 min, 4 ml of 7.5% (w/v) Na_2_CO_3_ prepared in distilled water was added to the mixture. The solution was mixed well and incubated at 20°C for 1 h. The absorbance was measured at 765 nm using DR 5000™ (Hach) spectrophotometer. A calibration curve (*y* = 0.0086*x* + 0.2546, *R* ^2^ = 0.9998) of gallic acid was prepared using solutions of varying concentrations ranging from 25 to 400 mg/L. Then, the amount of total phenolics present in the extract was measured in gallic acid equivalents (GAE) using the formula: A = (C × V)/m, where, A is the total amount of phenolics equivalent to gallic acid present in the extract, C is the concentration of gallic acid (mg/ml) measured from the calibration curve, V is the extract volume (ml) and m denotes extract weight (g). The process was conducted in triplicate, and the mean value of TPC was determined.

### 2.7 Determination of total flavonoid content (TFC)

A solution (1 ml) of extract (200 μg/ml) was taken in a test tube, and 2 ml of MeOH was added to it. Then the solution was mixed well with 0.1 ml of 10% of aluminum chloride (w/v, prepared in distilled water) followed by 1 M of Na‐K tartrate, 2.8 ml of distilled water, and incubated at 25°C. After 30 min, the absorbance of the mixture was measured at 415 nm (Selim et al., 2014). The calibration curve of quercetin (*y* = 0.0178*x* + 0.6152, *R* ^2^ = 0.9975) was prepared by measuring the absorbance of its different concentrations (25–400 mg/L). Then, the total flavonoid content of the extract was calculated using the standard calibration curve and expressed as mg of flavonoid present per gm of extract equivalent to quercetin. The experiment was conducted three times, and the mean value of flavonoid content was calculated.

### 2.8 Antioxidant activity test

#### 2.8.1 DPPH free radical scavenging capacity assay

The effect of MEPS on free radicals was determined by analyzing its scavenging effect on stable 1,1‐diphenyl‐2‐picrylhydrazyl (DPPH) free radicals. The plant extract or standard drug (ascorbic acid) was prepared at a concentration ranging from 400 to 1.5625 μg/ml in MeOH. A 0.1 mM solution of DPPH in MeOH was prepared, and 2 ml of this solution was added to 2 ml of the test solution. The mixture was mixed properly and incubated for 30 min at room temperature in a dark place. The absorbances for the standard and experimental solutions were measured against blank (without test sample or drug) DPPH solution using a spectrophotometer at 517 nm (Wang et al., 2013). The scavenging of DPPH free radicals was expressed as a percentage of inhibition was determined from the following equation:

$$\%\text{inhibition}=\frac{\left(\text{absorbance of blank}-\text{absorbance of test sample}\;\right)}{\text{absorbance of blank}}\times 100$$

then, IC_50_ value was calculated from % inhibition vs log concentration curve.

#### 2.8.2 Nitric oxide (NO) scavenging capacity assay

Exactly 4 ml of MEPS or standard (ascorbic acid) solution at the concentration 400–1.5625 μg/ml in methanol was taken in different test tubes. Then, 1.0 ml of sodium nitroprusside (5 mM) was added to the samples and incubated for 2 h at 30°C. After incubation, 2 ml solution was taken, and 1.2 ml Griess reagent (1% sulfanilamide, 0.1% napthylene diamine dihydrochloride in 2% H_3_PO_4_) was added to it. The absorbances for the standard and test solutions were measured against blank using a spectrophotometer at 550 nm (Alisi & Onyeze, 2008). The percentage of inhibition was calculated as described earlier in DPPH free radical scavenging assay, and the IC_50_ value was calculated.

### 2.9 Study animals

Swiss albino mice of either sex, weighing 25–30 g, 6–8 weeks, were used for the antihyperglycemic study. They were procured from the International Centre for Diarrhoeal Disease Research, Bangladesh (ICDDR, B) and housed in appropriate cages with wood flakes bedding. The mice were allowed to acclimatize for 2 weeks in standard laboratory conditions and were maintained at 25°C ± 2°C temperature, 55%–60% relative humidity, and 12 h light/dark cycle. They had access to water and feed ad libitum. The feed was formulated by authorities of ICCDR,B. The animals were randomly divided into five groups (normal control, diabetic control, and three experimental groups), each group consisting of five mice (*n* = 5). The normal and diabetic control groups received oral treatment of vehicle (physiological saline). The positive control and experimental groups were orally treated (p.o) with metformin and MEPS, respectively. The experimental mice starved from feed for 12 h but had free access to water before experiments. The tests were performed between 9.00 a.m. and 5.00 p.m., and the investigators had no information about the experimental groups.

### 2.10 Acute toxicity test

The acute toxic effect of MEPS on animals was assessed before studying the antihyperglycemic activity. Experimental animals were divided into four experimental and one control group (*n* = 5). The experimental animals were orally treated with MEPS at the doses of 500, 1000, 2000, and 3000 mg/kg b.w. Control group animals received physiological saline only. Animals were housed and adequately provided with ICCDR,B formulated food and water ad libitum. They were carefully observed for 72 h after administration of MEPS, and any adverse reactions (skin rashes, swelling, itching), behavioral changes, and mortality were documented (Walker et al., 2008).

### 2.11 Antihyperglycemic activity test

#### 2.11.1 Oral glucose tolerance test (OGTT)

The mice of control, diabetic control, standard drug treatment (positive control), and MEPS treatments (experimental groups) fasted overnight. The blood samples of each animal were collected from the tail vein, and glucose level was measured using Accu‐Chek® (Roche) one‐touch glucometer as baseline (0 min). Then animals of diabetic control, positive control, and experimental groups received vehicle (10 ml/kg b.w.), metformin (60 mg/kg b.w.), and MEPS (50, 150, 300 mg/kg b.w.), respectively. Primarily, antihyperglycemic activities were evaluated with the lower doses (50 mg/kg b.w.) of MEPS and the dose was randomly selected based on observing the effect of *P. sylvestris* fruits in the previous study (Shajib et al., 2015). The higher dose limit (300 mg/kg b.w.) was selected based on the significant glucose‐lowering effect of MEPS. After 30 min, each group of mice received 10% glucose solution at the dose of 2 gm/kg b.w. Then, blood glucose level was measured at 30, 60, 90, and 120 min following glucose treatment (Chaturvedi et al., 2004).

#### 2.11.2 Assay for alloxan‐induced diabetes

The experimental mice were randomly divided into control, diabetic control, standard drug treatment (positive control), and MEPS treatments (experimental groups). Positive control and experimental group animals were induced with diabetes by intraperitoneal (i.p.) injection of alloxan‐monohydrate at the dose of 60 mg/kg b.w. The blood glucose level was measured before alloxan treatment. The glucose levels were monitored every day after alloxan treatment. Alloxan induces type 1 or insulin‐dependent diabetes (Macdonald Ighodaro et al., 2017). In fasting conditions, blood glucose level of more than 7 mmol/L is indicative of diabetes (Adeyi et al., 2015; Mathew & Tadi, 2021; Njogu et al., 2016). After 3 days of alloxan administration, fasted mice with blood sugar levels ≥8 mmol/L were considered diabetic (Ezeja et al., 2015). The sustained hyperglycemia of the alloxan‐induced diabetic mice was observed for the next 5 days and selected for the study. Alloxan may increase blood glucose levels by more than 11 mmol/L in consecutive days after administration (Macdonald Ighodaro et al., 2017; Njogu et al., 2016). However, the time required to reach the blood glucose level can vary on the alloxan administration route, dose, and experimental animal species (Hansen et al., 2007; Kim et al., 2006; Lips et al., 1988; Njogu et al., 2016). The hyperglycemic mice received vehicle (10 ml/kg b.w.), metformin (60 mg/kg b.w.), or MEPS (50, 150, and 300 mg/kg b.w.). Blood samples were collected from the tail vein of each group of mice, and glucose level was measured at 0 h (as baseline), 4, 8, and 24 h following treatments (Semwal et al., 2010).

### 2.12 Statistical analysis

All the experimental data were presented as mean ± SEM (standard error of the mean). IC_50_ values were determined by utilizing GraphPad Prism 6.01 (GraphPad Software, Inc.). The comparison of different groups against the control group was performed by one‐way analysis of variance (ANOVA) followed by Dunnett's test as the post hoc test using SPSS 22 (IBM) software. *p* < .05 was set as the level of statistical significance.

## 3 RESULTS

### 3.1 Phytochemical analysis

**FIGURE 1:**
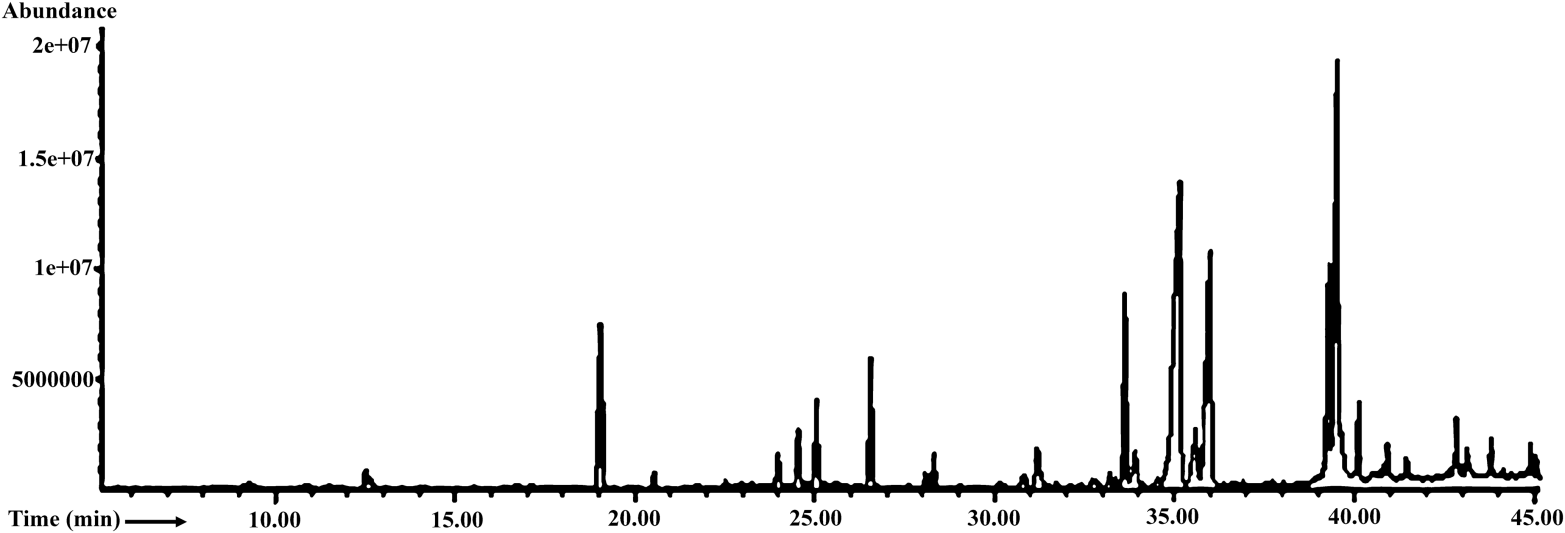
Gas‐chromatographic fingerprint of methanol extract of *P. sylvestris* seeds (MEPS). The chromatogram was obtained with the ionization potential of 70 eV.

Preliminary screening for different phytochemical groups reveals that the plant seed contained alkaloids, steroids, carbohydrates, proteins, flavonoids, and tannins. The most abundant compound revealed by the GC–MS analysis of the extract was 8‐methylisoquinoline N‐oxide (32.82%). Other major constituents were as follows: methyl oleate (12.19%), methyl linoleate (7.44%), dodecanoic acid, methyl ester (5.59%), palmitic acid, methyl ester (4.62%), 9‐octadecenoic acid (Z)‐,2,3‐dihydroxypropyl ester (3.11%), 5,8‐dimethyl‐1,4‐dihydro‐1,4‐methanonaphthalene (2.93%), tetradecanoic acid, methyl ester (2.88%), alpha‐bisabolol (2.41%),linalool (1.69%), (+)‐(4 S, 8R)‐8‐epi‐beta‐bisabolol (1.59%), 1‐fluoro‐4‐acetylbenzene (1.56%), methyl stearate (1.41%), 11‐eicosenoic acid, methyl ester (1.39%), and alpha‐bisabolol oxide B (1.15%). The identified compounds, peak area (%), and retention time (min) of MEPS by GC–MS analysis are presented in Table 1. The total ionic chromatograph of the methanol extract of *P. sylvestris* seed is shown in Figure 1.

**TABLE 1:** Constituents of methanol extract of *Phoenix sylvestris* seeds identified by GC–MS analysis

Serial No.	Retention time	Peak area (%)	Constituent
1	9.287	0.49	3‐Methylquinoline‐1‐oxide
2	10.860	0.92	1‐Ethyl‐2‐methyl‐cyclopentane
3	12.508	0.95	Alpha‐cubebene
4	19.054	5.59	Dodecanoic acid, methyl ester
5	19.849	0.61	3,4‐Dihydro‐8‐hydroxy‐3‐methylisocoumarin
6	20.542	0.89	Nerolidol
7	23.574	0.48	d‐Mannitol
8	23.986	1.15	Alpha‐bisabolol oxide B
9	24.541	1.59	(+)‐(4 S,8R)‐8‐epi‐beta‐bisabolol
10	25.051	2.41	Alpha‐bisabolol
11	25.623	0.37	Ribitol
12	26.561	2.88	Tetradecanoic acid, methyl ester
13	28.083	0.55	Methyl 4‐hydroxybenzoate
14	28.312	32.82	8‐Methylisoquinoline N‐oxide
15	28.467	0.36	1‐Butylisoquinoline
16	30.853	0.44	Nerolidoloxide
17	31.185	1.69	Linalool
18	31.728;	0.55	Bisabolol oxide A
19	32.701	0.36	1,4‐Dihydro‐3H‐2‐Benzopyran‐3‐imine
20	32.861	0.68	9‐Hexadecenoic acid, methyl ester, (Z)‐
21	33.634	4.62	Palmitic acid, methyl ester
22	33.903	1.56	1‐Fluoro‐4‐acetylbenzene
23	34.555	0.39	(+/−)‐Citronellol
24	35.591	2.93	5,8‐Dimethyl‐1,4‐dihydro‐1,4‐methanonaphthalene
25	36.374	0.23	Geranyl isovalerate
26	36.615	0.31	Phytol
27	36.724	0.65	Isocyclocitral
28	39.287	7.44	Methyl linoleate
29	39.516	12.19	Methyl oleate
30	39.619	3.11	9‐Octadecenoic acid (Z)‐, 2,3‐dihydroxypropyl ester
31	40.117	1.41	Methyl stearate
32	40.477	0.70	6‐Octadecenoic acid
33	40.557	0.66	3‐Phenyl‐1,4(E)‐dodecadiene
34	40.929	0.89	Ethyl oleate
35	41.250	0.98	Cis‐vaccenic acid
36	41.747	0.98	Oleic acid
37	42.640	0.39	Geranyl‐geraniol
38	42.840	1.39	11‐Eicosenoic acid, methyl ester
39	43.144	0.97	Eicosanoic acid, methyl ester
40	43.807	0.91	Squalene
41	44.900	0.89	2‐Monopalmitin
42	45.060	0.51	Methyl behenate

### 3.2 Antioxidant activity

**FIGURE 2:**
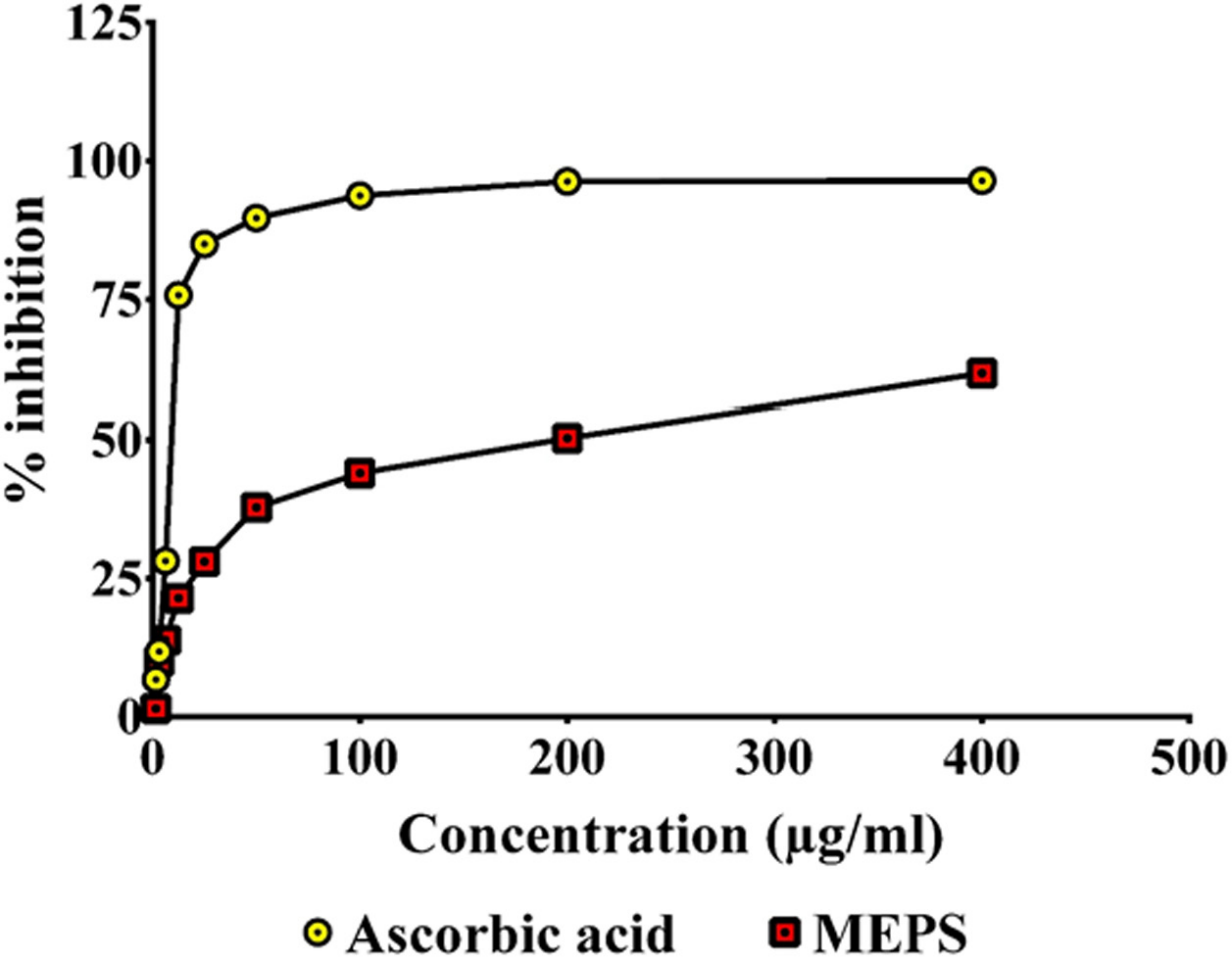
DPPH (2,2‐diphenyl‐1‐picrylhydrazyl) free radicals scavenging activity of ascorbic acid (standard) and MEPS

**FIGURE 3:**
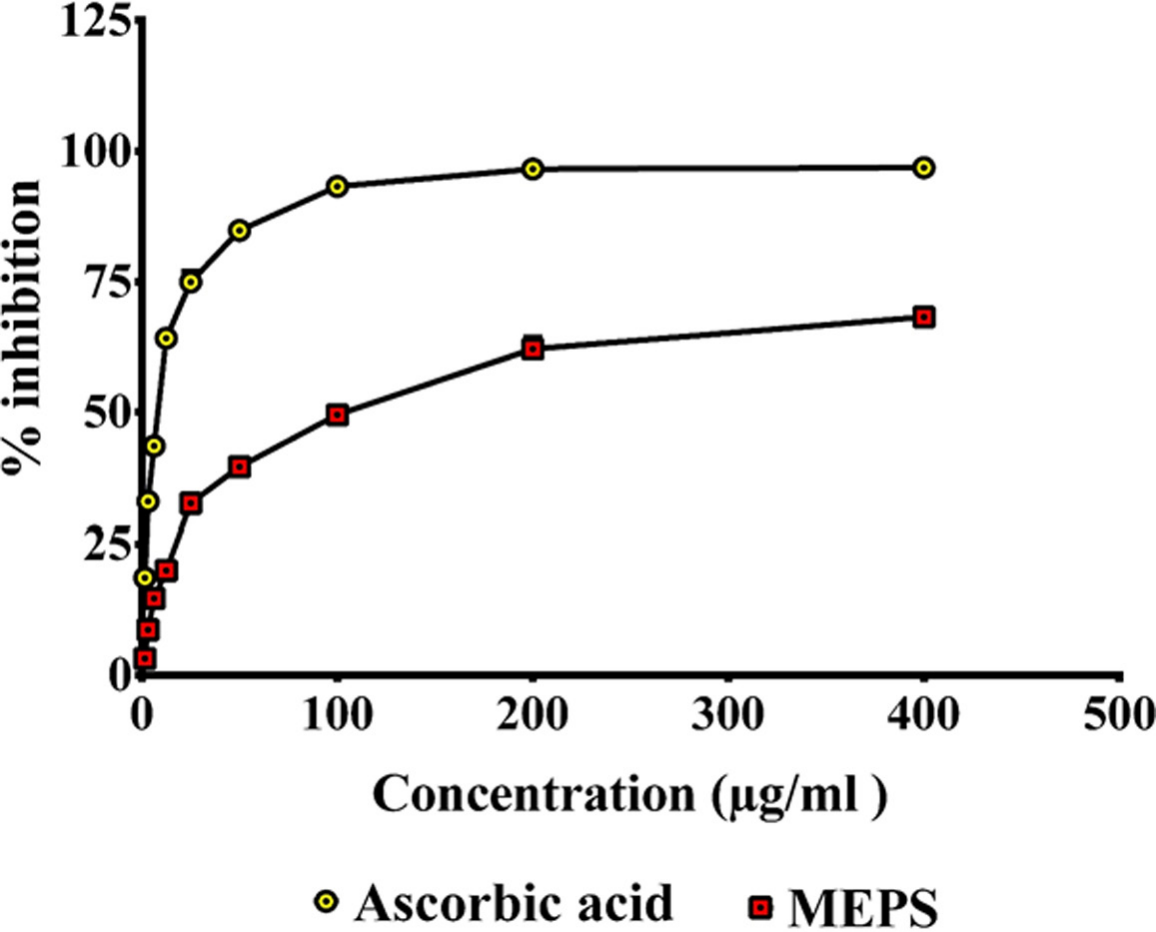
Nitric oxide (NO) free radicals scavenging activity of ascorbic acid (standard) and MEPS

Quantitative analysis of the crude extract demonstrated that there are 91.32 ± 5.20 mg total phenolics equivalent to gallic acid and 21.99 ± 4.70 mg total flavonoids equivalent to quercetin present in per gram extract. The anti‐radical activity of MEPS against DPPH and NO was found to have IC_50_ values of 162.70 ± 14.99 and 101.56 ± 9.46 μg/ml, respectively. Standard drug ascorbic acid demonstrated IC_50_ values of 8.71 ± 0.02 and 7.39 ± 0.43 μg/ml, respectively. The highest percent inhibition of DPPH radical exhibited by MEPS was 61.67 ± 1.74 at the maximum experimental concentration (400 μg/ml). Ascorbic acid inhibited DPPH radical by 96.41 ± 0.00% (Figure 2). MEPS and ascorbic acid displayed a maximum of 68.20 ± 1.00 and 96.78 ± 0.38% nitric oxide (NO) scavenging activity at higher concentrations, respectively (Figure 3). The results show that MEPS is capable of arresting the free radicals generated by DPPH and NO, which are harmful to human health (Hasan et al., 2009). It has been reported that plant phenolics and flavonoids may exert significant antioxidant activities (Rice‐Evans et al., 1997; Saija et al., 1995). The presence of a considerable amount of phenolics and flavonoids in MEPS can be attributed to its strong antioxidant activity.

### 3.3 Acute toxicity

Oral administration of MEPS up to 3000 mg/kg did not cause any adverse reactions, behavioral changes, or mortality during the observational period. This suggests that MEPS possesses a low toxicity profile (LD_50_ > 3000 mg/kg b.w.). The doses of the MEPS for antihyperglycemic studies were selected from trial experiments. The observations from the acute toxicity study indicate that the experimental doses of MEPS selected for the study were safe.

### 3.4 Oral glucose tolerance

**FIGURE 4:**
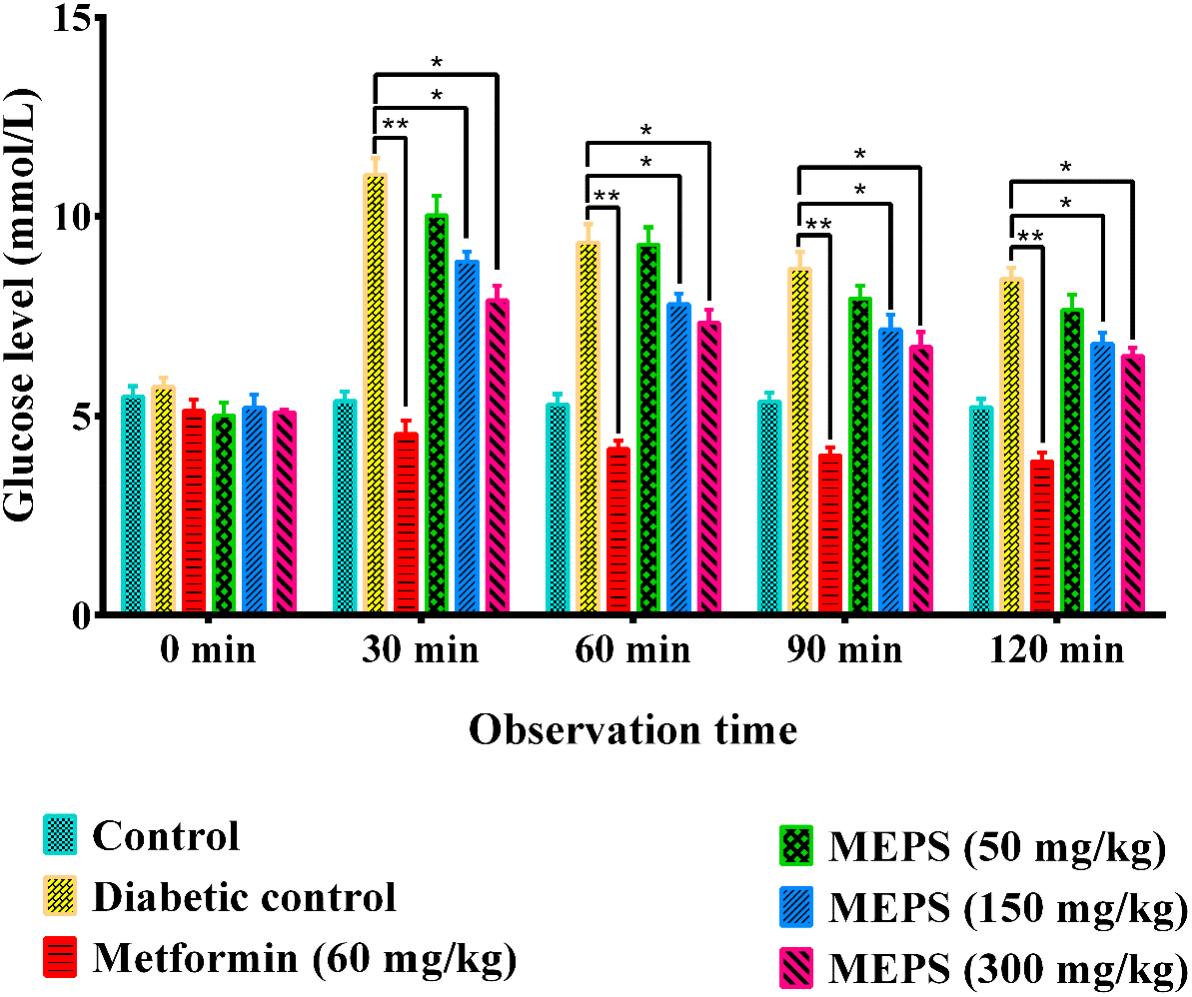
Effect of MEPS and metformin in oral glucose tolerance test. Data are expressed as mean ± SEM (*n* = 5). MEPS = methanol extract of *P. sylvestris* seeds. **p < .001 and *p < .05, compared to diabetic control group (Dunnett's test)

Oral glucose tolerance test (OGTT) measures the ability to utilize sugars by the body and is commonly performed to evaluate pre‐diabetes, post‐diabetes, and gestational diabetes (Hartling et al., 2012; Ziegler et al., 2009). The additional glucose load causes the excess plasma glucose level, characterized as hyperglycemia and early clinical manifestation of diabetes. Fasted mice showed glucose levels below 5.5 mmol/L, which was in the normal range (Andrikopoulos et al., 2008). After 30 min of oral glucose treatment, the plasma glucose level was significantly increased in mice and then gradually declined throughout the observation period. Oral treatment of MEPS and the standard drug metformin caused a marked reduction of the elevated blood glucose level in OGTT (Figure 4). The result was significant over the observation period (30–120 min) for both metformin (60 mg/kg b.w.) and MEPS at the doses of 150 and 300 mg/kg b.w. The rate of plasma glucose level reduction of MEPS was dose dependent. The result indicates that MEPS may exert protective action against the hyperglycemic condition of diabetes mellitus.

### 3.5 Alloxan‐induced diabetes

Oral ingestion of MEPS (150, 300 mg/kg b.w.) and standard drug metformin (60 mg/kg b.w.) exhibited significant (*p* < .001) antihyperglycemic effect in alloxan‐induced diabetic mice throughout the experimental period as shown in Table 2. Intraperitoneal treatment of alloxan (60 mg/kg b.w.) caused marked increases in glucose levels in the mice compared to the vehicle treatment group. The blood sugar level was steady at different measurement times from 0 to 24 h by nearly15 mmol/l for the alloxan‐induced diabetic control mice. The standard drug metformin significantly reduced the blood glucose level after 4 h of treatment (10 mg/kg b.w., p.o). The glucose level of alloxan‐induced diabetic mice also started to decline significantly following 4 h of oral treatment of MEPS at lower doses (50 mg/kg) compared to the diabetic control mice. However, the antihyperglycemic effect of MEPS was noticeably different from the metformin‐treated diabetic mice. The glucose‐lowering effect of MEPS was highest at the maximum dose (300 mg/kg) after 24 h of oral treatment.

**TABLE 2:** Effect of MEPS and metformin on alloxan‐induced diabetic mice

Group	Treatment	Blood glucose level (mmol/L)			
		0 h	4 h	8 h	24 h
Control	Vehicle (10 ml/kg)	5.25 ± 0.45	5.44 ± 0.39	5.19 ± 0.41	5.31 ± 0.45
Diabetic control	Vehicle (10 ml/kg)	15.48 ± 0.39	15.04 ± 0.46	14.97 ± 0.59	15.46 ± 0.19
Positive control	Metformin (10 mg/kg)	15.15 ± 0.59	6.88 ± 0.30*	4.97 ± 0.07*	4.46 ± 0.16*
Experimental 1	MEPS (50 mg/kg)	15.14 ± 0.42	13.83 ± 0.30*	13.73 ± 0.35	13.07 ± 0.43*
Experimental 2	MEPS (150 mg/kg)	15.33 ± 0.34	12.47 ± 0.37*	12.09 ± 0.35*	11.08 ± 0.33*
Experimental 3	MEPS (300 mg/kg)	15.85 ± 0.23	11.54 ± 0.36*	10.76 ± 0.21*	9.76 ± 0.28*

*Note*: Data are expressed as mean ± SEM (*n* = 5).

Abbreviation: MEPS, methanol extract of *P. sylvestris* seeds.

* *p* < .001, compared to diabetic control group (Dunnett's test).

## 4 DISCUSSION

Plants are gifts of nature housed thousands of important biochemical playing major roles in the regulation and maintenance of body's homeostasis (Alam et al., 2020, 2021; Islam et al., 2022). The present study investigates the antihyperglycemic activities of crude extract of *P. sylvestris* seed (MEPS) and the rationale for its use in diabetes as claimed in traditional medicine. The plant *P. sylvestris* is grown in the wild and cultivated in different regions of southeast Asia, including Bangladesh (Lamia & Mukti, 2021). The plant is also economically valued for its multiple households, industrial purposes, and nutritional and medicinal significance in Bangladesh (Chowdhury et al., 2008; Lamia & Mukti, 2021). Previous studies reported that the plant seeds are enriched with antioxidants (Kothari et al., 2012) and protective oil (Qidwai et al., 2018). Recently published literature demonstrated that the alcohol extract seed of *Phoenix dactilyfera*, a native date palm of the Arecaceae family, possess promising free radical scavenging and reduced blood glucose in diabetic rats (Abiola et al., 2018). The current study reveals the phytochemicals possibly responsible for oxidative radical scavenging capacity and antihyperglycemic activities of *P. sylvestris* seed extensively grown in Bangladesh.

The plant extracts and compounds with profound antioxidant capacity could be promising candidates for the management of recovery of oxidative stress‐induced diseases such as diabetes (Ashrafi et al., 2022; Sultana et al., 2022; Vinayagam et al., 2016). The phenolics and flavonoids are the significant phytochemicals evidenced to remarkably restore oxidative damage by scavenging free radicals produced in diabetic patients (Emon et al., 2020, 2021; Sarian et al., 2017; Vinayagam et al., 2016). Pre‐clinical studies showed that plant phenolics could elevate plasma insulin levels and increase glucose uptake by accelerating hepatic glycolysis, glucogenesis, and gluconeogenesis (Chakrabarty et al., 2022; Rudra et al., 2020; Vinayagam et al., 2016). The antioxidant defense mechanism of flavonoids involves the mitigation of reactive oxidative species‐induced endothelial cell damage and endoplasmic reticulum stress responsible for impaired insulin and hyperglycemia (Sarian et al., 2017). The presence of a substantial amount of total phenolic and flavonoid contents and the prominent antioxidant capacity of the crude extract of *P. sylvestris* seed has been evidenced in the recently published literatures (Kothari et al., 2012; Qidwai et al., 2018). However, it was noticeable that the phytochemical contents varied with the extraction methods (Kothari et al., 2012; Qidwai et al., 2018). The variability could also be responsible for the geographical, ecological, and botanical conditions and harvesting times. The result of the present study indicates *P. sylvestris* seed (MEPS) grown in Bangladesh contains substantial amounts of phenolics and flavonoids. The results showed that the scavenging of DPPH and NO free radicals by MEPS was also noticeable. Furthermore, several antioxidant compounds, including nerolidol (Neto et al., 2013), citronellol (Jagdale et al., 2015), and phytol (Santos et al., 2013) were identified from the GC–MS analysis of MEPS. The substantial retention of phenolics, flavonoid compounds, and promising free radicals detaining capacity of MEPS further encouraged to proceed the investigation of its effect against oxidative stress‐related hyperglycemia.

Hyperglycemia and fluctuation of blood glucose levels are critical pathological indicators of the development and progression of diabetes (Mathew & Tadi, 2021). Oral glucose tolerance test primarily indicates the impairment of glucose tolerance indicates insulin resistance and associated problems of carbohydrate metabolism (Andrikopoulos et al., 2008). The test is also commonly performed to evaluate the glucose tolerance improvement capability of drug candidates or plant extracts before assessment into the additional diabetic model (Abiola et al., 2018; Dauki et al., 2022; Sornalakshmi et al., 2016). In glucose‐ingested non‐diabetic mice, MEPS treatment showed a significant reduction in plasma glucose level. The result indicates that MEPS could be effective for the improvement of metabolic uptake of glucose and re‐establish the normal blood glucose level. To justify the enhancement of glucose tolerance in diabetic‐associated condition, MEPS was further challenged in alloxan‐induced diabetic mice. Alloxan selectively causes damage to a large number of pancreatic beta cells, inhibiting the sensitivity of pancreatic glucokinase enzyme, which results in reduced insulin release and glucose uptake by the tissues. Therefore, the glucose level of blood is significantly raised, and the consequence is characterized as hyperglycemia (Saravanan & Pari, 2005). Besides, alloxan administration induces excessive generation of free radicals such as reactive oxygen species (ROS) by the activation of hydroperoxides, and lipid peroxidation system, which leads to pancreatic tissue injury as well as promotes the pathogenic consequences of diabetes (Halliwell & Gutteridge, 2015; Sabu & Kuttan, 2004). Both mechanisms of alloxan action lead to a pathological state of type 1‐like diabetes or insulin‐dependent diabetes in cells (Macdonald Ighodaro et al., 2017). The significant decrease in blood level by the MEPS (Table 2) indicates that it remarkably alleviated the hyperglycemic effect produced by alloxan. Its antioxidant potential may play a pivotal role in the effects. The presence of antidiabetic agent linalool (More et al., 2014) as well as antioxidant compounds nerolidol (Neto et al., 2013), citronellol (Jagdale et al., 2015) and phytol (Santos et al., 2013) in MEPS (Table 1) further supports the outcome of the study.

## 5 CONCLUSION

The present study revealed that the methanol extract of *P. sylvestris* (MEPS) possesses strong antioxidant and antihyperglycemic activities. Quantitative analysis of MEPS indicated that it contains a considerable amount of phenolics and flavonoids. Besides, MEPS showed potent scavenging activity against the free radicals generated by DPPH and NO. MEPS significantly reduced the hyperglycemic effect induced by glucose and alloxan. This effect could be associated with its antioxidant action as well as the presence of the bioactive compounds, which were confirmed by GC–MS analysis. Therefore, further studies on the isolation as well as analysis of the biological activities of the isolated compounds, are required. The results of the present study indicate that *P. sylvestris* seed could be a potential natural source for developing antidiabetic compounds.

## FUNDING INFORMATION

The investigation was partially done in the Molecular Pharmacology and Herbal Drug Research Laboratory, which was established through financial support from the Higher Education Quality Enhancement Project (HEQEP), AIF, Round‐III, Window‐2, CP‐3258, University Grants Commission (UGC) of Bangladesh.

## CONFLICT OF INTEREST

No potential conflict of interest was reported by the authors.

## Data Availability

All analyzed data during this research are included in the published manuscript. The generated datasets during this research are not publicly available, although they can be provided from the corresponding author upon reasonable request.
